# Significant Therapeutic Effects of Adult Human Neural Stem Cells for Spinal Cord Injury Are Mediated by Monocyte Chemoattractant Protein-1 (MCP-1)

**DOI:** 10.3390/ijms23084267

**Published:** 2022-04-12

**Authors:** Chung Kwon Kim, Jeong-Seob Won, Jae Yeol An, Ho Jin Lee, Ah-Jin Nam, Hyun Nam, Ji Yeoun Lee, Kyung-Hoon Lee, Sun-Ho Lee, Kyeung Min Joo

**Affiliations:** 1 Medical Innovation Technology Inc. (MEDINNO Inc.), Ace High-End Tower Classic 26, Seoul 08517, Korea; kimck0405@gmail.com (C.K.K.); wjdtjq1124@gmail.com (J.-S.W.); snutaeng@gmail.com (H.N.); 2 Biomedical Institute for Convergence at SKKU (BICS), Sungkyunkwan University, Suwon 16419, Korea; 3 Stem Cell and Regenerative Medicine Institute, Research Institute for Future Medicine, Samsung Medical Center, Seoul 06351, Korea; 4 Single Cell Network Research Center, Sungkyunkwan University School of Medicine, Suwon 16419, Korea; hry0608@gmail.com (H.J.L.); leekh@skku.edu (K.-H.L.); 5 Department of Health Sciences and Technology, SAIHST, Sungkyunkwan University, Seoul 06351, Korea; 6 Department of Anatomy, Seoul National University College of Medicine, Seoul 03880, Korea; jaeyeol.an@partnersi.co.kr (J.Y.A.); ddang1@snu.ac.kr (J.Y.L.); 7 Healthcare Division, Partners Investment Co., Ltd., Seoul 06152, Korea; 8 Department of Anatomy & Cell Biology, Sungkyunkwan University School of Medicine, Suwon 16419, Korea; nag1201@naver.com; 9 Department of Neurosurgery, Samsung Medical Center, Sungkyunkwan University School of Medicine, Seoul 06351, Korea; 10 Division of Pediatric Neurosurgery, Seoul National University Children’s Hospital, Seoul 03080, Korea

**Keywords:** spinal cord injury, neural stem cell, dose escalation, lateral ventricle, monocyte chemoattractant protein-1

## Abstract

The limited capability of regeneration in the human central nervous system leads to severe and permanent disabilities following spinal cord injury (SCI) while patients suffer from no viable treatment option. Adult human neural stem cells (ahNSCs) are unique cells derived from the adult human brain, which have the essential characteristics of NSCs. The objective of this study was to characterize the therapeutic effects of ahNSCs isolated from the temporal lobes of focal cortical dysplasia type IIIa for SCI and to elucidate their treatment mechanisms. Results showed that the recovery of motor functions was significantly improved in groups transplanted with ahNSCs, where, in damaged regions of spinal cords, the numbers of both spread and regenerated nerve fibers were observed to be higher than the vehicle group. In addition, the distance between neuronal nuclei in damaged spinal cord tissue was significantly closer in treatment groups than the vehicle group. Based on an immunohistochemistry analysis, those neuroprotective effects of ahNSCs in SCI were found to be mediated by inhibiting apoptosis of spinal cord neurons. Moreover, the analysis of the conditioned medium (CM) of ahNSCs revealed that such neuroprotective effects were mediated by paracrine effects with various types of cytokines released from ahNSCs, where monocyte chemoattractant protein-1 (MCP-1, also known as CCL2) was identified as a key paracrine mediator. These results of ahNSCs could be utilized further in the preclinical and clinical development of effective and safe cell therapeutics for SCI, with no available therapeutic options at present.

## 1. Introduction

Axonal regeneration from injured neurons hardly occurs in the adult mammalian central nervous system (CNS) [1]. Such a low neuron-intrinsic regenerative capacity in the CNS is mainly attributed to the inhibitory microenvironment of glial scar, which generates chondroitin sulfate proteoglycans (CSPGs) [2]. The limited regenerative potential of the CNS results in severe and permanent disabilities after spinal cord injury (SCI), such as motor paralysis and neuropathic pain [3,4]. As of now, no clinically meaningful treatments are available for SCI to reverse the persistent sequalae, emphasizing the need for novel therapeutic strategies enabling functional recovery in SCI patients [5].

Neural stem cell (NSC) therapies have been studied in various neurological pathologies as an alternative option [6,7]. Several pre-clinical studies have shown NSCs to be safe and effective, leading to an increasing number of clinical trials [8,9,10]. NSC transplantation can provide feasible benefits through various mechanisms in the damaged CNS, such as modulation of the inflammatory response, paracrine neuroprotective effects, and regeneration of lost neural tissue [11,12]. Previously, we transplanted adult human NSCs (ahNSCs) into the lateral ventricle (LV) of SCI animal models and observed significant therapeutic effects [13]. The ahNSCs injected into the LV migrated towards damaged regions of the spinal cord and reduced excessive glial scar formation. We also suggested that their paracrine factors might also provide neuroprotective effects and promote angiogenesis [13]. It is important to note that, however, the surgical tissue of patients with hemorrhagic stroke is technically difficult to obtain. Moreover, the paracrine factors that mediated beneficial therapeutic effects were poorly identified in the previous study.

To overcome those limitations, recently, we demonstrated that ahNSCs derived from focal cortical dysplasia (FCD) type IIIa have cellular properties like neural stem/precursor cells, including a self-renewing and neural-differentiation potency [14,15,16,17,18]. In this study, we analyzed the therapeutic effects of ahNSCs isolated from temporal lobes of focal cortical dysplasia type IIIa for SCI and to elucidate their treatment mechanisms. Additionally, we identified neuroprotective effects of monocyte chemoattractant protein-1 (MCP-1; also known as CCL2) among various cytokines released from ahNSCs.

## 2. Results

### 2.1. Therapeutic Effect of ahNSCs for SCI

**Figure 1:**
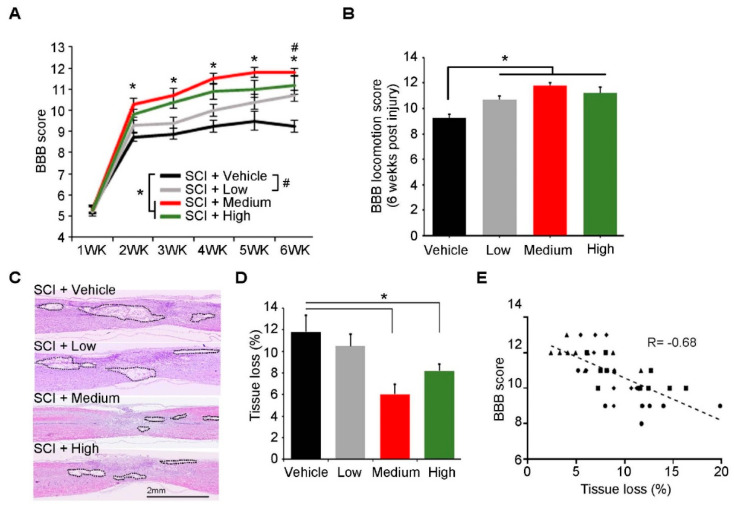
Preclinical therapeutic effects of ahNSCs for SCI. (**A**) BBB scores were measured and compared once a week until 6 weeks after SCI. *, #, *p* < 0.05. (**B**) BBB scores were measured and compared at 6 weeks after SCI. *, *p* < 0.05. (**C**) Tissue loss at the injury site was evaluated at 6 weeks post-SCI. Black lines delineate the contours of cavities and shrunken tissue. Scale bar = 2 mm. (**D**) Relative areas of tissue loss were quantified and compared. *, *p* < 0.05. (**E**) A correlation analysis revealed a significant negative relationship between the degree of tissue loss and the BBB score at 6 weeks post-SCI. Each dot represents two variables for each individual (●: vehicle group, ■: low group, ▲: medium group, ♦: high group, respectively).

To examine the therapeutic effects of ahNSCs for SCI, 3 × 10^5^ (low), 1 × 10^6^ (medium), or 3 × 10^6^ (high) ahNSCs in 30 μL Hanks’ Balanced Salt solution (HBSS) were transplanted into the LV of SCI animal models at 1 week after injury (*n* = 8 for each group). When recovery of motor functions was measured by the Basso, Beattie, and Bresnahan (BBB) score (Supplementary Table S1), the medium and high groups showed significantly higher scores compared to the vehicle group (30 μL HBSS, *n* = 10) from 1 week to 5 weeks after the treatment (Figure 1A,B). However, significant differences were not observed among the three groups transplanted with ahNSCs. The low group had a statistically significant functional recovery only at 5 weeks after the transplantation of ahNSCs.

When the area of cavity was measured in the damaged spinal cords at 5 weeks after treatment (Figure 1C), the medium and high groups showed significantly smaller cavity areas than that of the vehicle group (Figure 1D). In contrast, there was no significant difference between the low and vehicle group. The tissue loss of the medium group was significantly less than that of the low group. A significant negative correlation between the BBB score at 6 weeks post-SCI and the tissue loss in the spinal cord was observed in the analysis of the cavity size (Figure 1E). These results indicate that ahNSCs derived from the temporal lobe of FCD type IIIa surgical samples had significant therapeutic effects for SCI in a dose-dependent manner, which are similar to those of ahNSCs derived from the surgical samples of a hemorrhagic stroke [13].

### 2.2. In Vivo Neuroprotective Effects of ahNSCs

**Figure 2:**
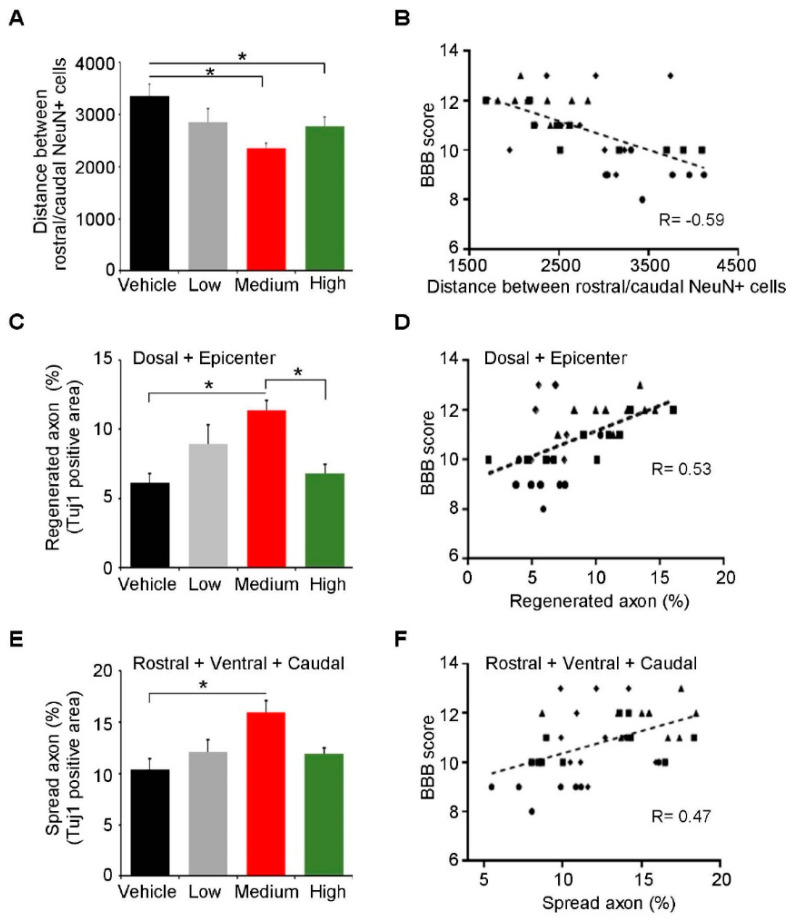
In vivo neuroprotective activities of ahNSCs. (**A**) NeuN-positive neurons adjacent to damaged lesions both rostrally and caudally were identified. The distance between those neurons was quantified and compared. (**B**) The correlation between the distance and the BBB score at 6 weeks post-SCI was analyzed. (**C**) Tuj1 immunoreactivity was evaluated in five areas of a damaged spinal cord: Dorsal, Epicenter, Rostral, Ventral, and Caudal. Tuj1 immunoreactive areas of the Dorsal and Epicenter areas were quantified and compared. (**D**) The correlation between the Tuj1 immunoreactivity of the Dorsal and Epicenter area and the BBB score at 6 weeks post-SCI was analyzed. (**E**) Tuj1 immunoreactive areas of Rostral, Ventral, and Caudal areas were quantified and compared among groups. (**F**) The correlation between the immunoreactivity of Tuj1 in the Rostral, Ventral, and Caudal area and the BBB score at 6 weeks post-SCI was analyzed. *, *p* < 0.05. Each dot represents two variables for each individual (●: vehicle group, ■: low group, ▲: medium group, ♦: high group, respectively).

To find he in vivo treatment mechanism of ahNSCs, immunohistochemistry against NeuN and Tuj1 was performed 5 weeks after transplantation. Since NeuN and Tuj1 are the specific markers of a viable neuronal cell body and nerve fiber of neurons, respectively, the distance between the rostral and caudal NeuN-positive neurons in a damaged spinal cord would indicate the degree of tissue degeneration by SCI. We observed that the distance of the vehicle group was significantly farther than those of the medium and high groups (Figure 2A and Supplementary Figure S1A). Moreover, the distance showed a significant negative correlation with the BBB score at 6 weeks post-SCI (Figure 2B).

Next, immunoreactivity against Tuj1 showed viable axons and dendrites at five points near the lesion (dorsal, epicenter, rostral, ventral, and caudal). Tuj1-positive nerve fibers at the dorsal and epicenter points would indicate the regeneration of fibers, given that the spinal cord tissue of those areas was directly destroyed by traumatic damage (regenerated axon) (Supplementary Figure S1B). On the other side, the degree of tissue damage could be indirectly evaluated by immunoreactivity against Tuj1 at the rostral, ventral, and caudal areas, since physical force was delivered to the spinal cord dorsally (spared axon) (Supplementary Figure S1C). At both analyses, Tuj1 expression of the medium group was significantly higher than the vehicle group (Figure 2C,E). Meanwhile, the high groups showed significantly less degeneration of the neuronal nuclei compared to the vehicle group (Figure 2B); a significant difference of Tuj1 expression between the high and vehicle group was not observed (Figure 2C,E). High Tuj1 expression was significantly correlated with a high BBB score at 6 weeks post-SCI in both analyses (Figure 2D,F).

**Figure 3:**
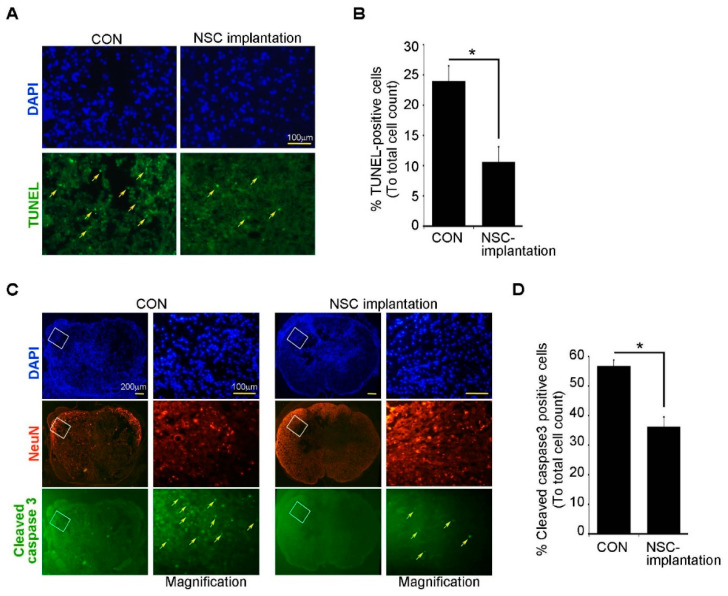
In vivo neuroprotective effects of ahNSCs. (**A**) Representative images of the TUNEL assay in the spinal cords of SCI animal models (**B**) TUNEL-positive cells were counted in each section and compared among groups (*n* = 5 per group). *, *p* < 0.05. (**C**) Representative images of cleaved caspase 3-positive neurons (NeuN^+^) in the spinal cords of SCI animal models (**D**) The number of cleaved caspase 3-positive cells was counted in each section and compared among groups (*n* = 5 per group). *, *p* < 0.05.

Taken together, these results indicate that ahNSCs mediated the neuroprotective effects in the injured spinal cords and showed significant therapeutic effects. To validate the neuroprotective effects of ahNSCs further, an apoptosis of neural cells in damaged spinal cords was analyzed at 7 days after treatment of 1 × 10^6^ ahNSCs (*n* = 5). It was observed that the transplantation of ahNSCs significantly reduced terminal deoxynucleotidyl transferase dUTP nick end labeling (TUNEL)-positive apoptotic cells (Figure 3A,B). Moreover, NeuN- and cleaved caspase 3-double positives significantly decreased in the ahNSCs transplanted group compared to the vehicle group (30 μL HBSS, *n* = 5) (Figure 3C,D), indicating that the neuroprotective effects of ahNSCs on spinal cord neurons might be mediated by inhibiting apoptosis.

### 2.3. In Vitro Neuroprotective Effects of ahNSCs

**Figure 4:**
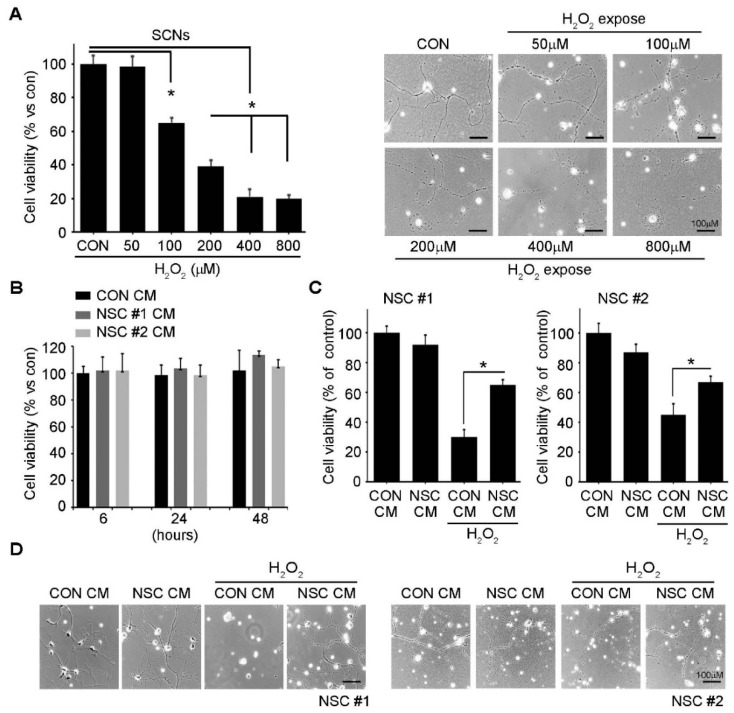
In vitro neuroprotective effects of ahNSCs. (**A**) The survival rate of SCNs treated with various concentrations of H_2_O_2_ for 24 h was measured by an MTT assay (*n* = 5 for each group). *, *p* < 0.05. Representative images of SCNs were illustrated. Scale bar = 100 μm. (**B**) An MTT assay was performed to estimate the viability of SCNs treated with the CM of NSC #1 or NSC#2. (**C**) SCNs were treated with the CM of NSC #1 or NSC #2 for 1 h and then with 200 μM of H_2_O_2_ for 24 h. The viability of SCNs was evaluated by an MTT assay (*n* = 5 for each group). *, *p* < 0.05. (**D**) Representative images of SCNs were illustrated. Scale bars = 100 μm.

The in vivo neuroprotective effects of ahNSCs were confirmed in vitro. When primary cultured rat spinal cord neurons (SCNs) were treated by various concentrations of H_2_O_2_ for 24 h, it was observed that the survival of SCNs was significantly reduced (Figure 4A). Moreover, the length of neurites of SCNs decreased dramatically by applying the H_2_O_2_ at 7 days in vitro (DIV 7) (Figure 4A). In contrast, the conditioned media (CM) of two independent batches of ahNSCs (NSC #1 and #2) did not cause similar toxic effects mediated by H_2_O_2_ on the survival and neurite length of SCNs (Figure 4B,D). When SCNs were maintained in the CM of ahNSCs for 1 h before H_2_O_2_ treatment, the toxic effects mediated by H_2_O_2_ were significantly reduced, and two batches of the CM of ahNSCs showed similar in vitro neuroprotective effects (Figure 4C,D). These results indicate that ahNSCs exert their neuroprotective effects in the paracrine manner.

**Figure 5:**
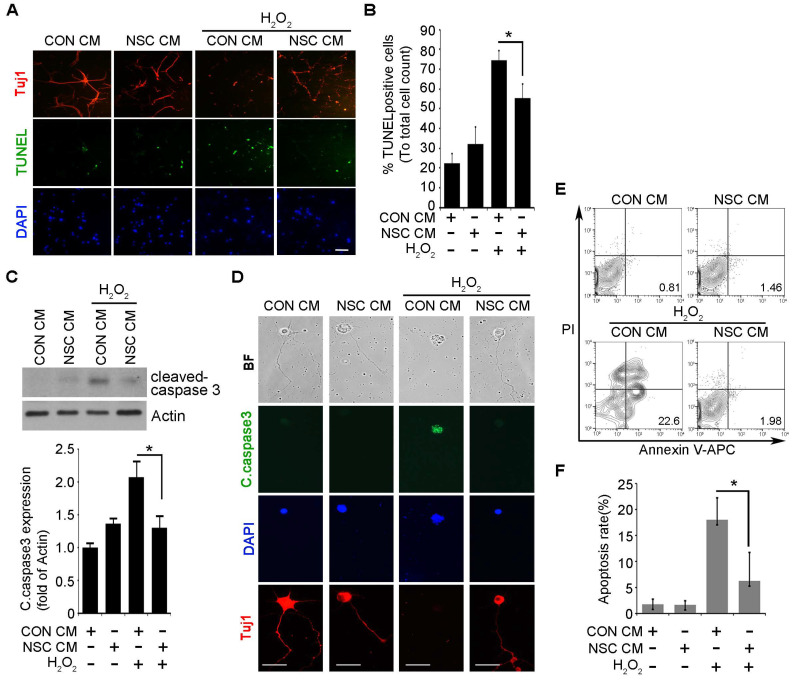
In vitro anti-apoptotic effects of ahNSC CM. (**A**) SCNs were treated with the CM of NSC #1 for 1 h and then with 200 μM of H_2_O_2_ for 24 h. TUNEL^+^ and/or Tuj1^+^ SCNs were visualized in each group. Scale bars = 100 μm. (**B**) TUNEL^+^ apoptotic SCNs were counted and compared (*n* = 5 per group). *, *p* < 0.05. (**C**) The expression of cleaved caspase 3 in each group was analyzed by a western blot analysis. Actin = loading control. The amount of cleaved caspase was quantified by densitometry analysis (normalized to actin) and compared (*n* = 3 per group). *, *p* < 0.05. (**D**) Tuj1 or cleaved caspase 3-postive SCNs were visualized in each group. Scale bars = 50 μm. (**E**) SCN cells were analyzed by Annexin-V/PI. (**F**) The apoptosis ratio was calculated from the percentage of early apoptosis among different experimental groups (*n* = 5 per group). *, *p* < 0.05.

**Figure 6:**
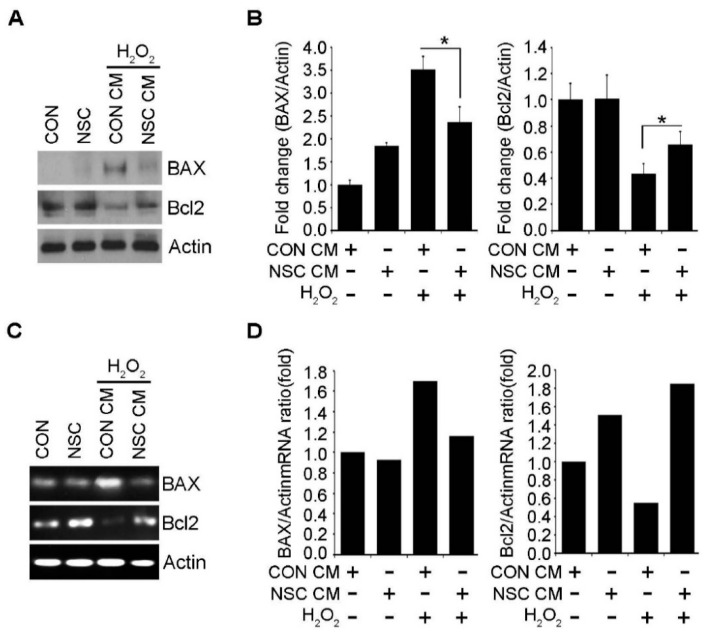
Apoptosis-related gene expression in SCN cells treated with ahNSC CM. (**A**) SCNs were treated with the CM of ahNSC #1 for 1 h and with 200 μM of H_2_O_2_ for 24 h. The expression of BAX and Bcl2 in each group was analyzed by a western blot analysis. Actin = loading control. (**B**) The amount of Bax and Bcl2 was quantified by a densitometry analysis (normalized to actin) and compared (*n* = 3 per group). *, *p* < 0.05. (**C**) The expression of BAX and Bcl2 in each group was analyzed by RT-PCR. Actin = internal control. (**D**) The amount of mRNA of Bax and Bcl2 was quantified by a densitometry analysis (normalized to actin) and compared.

When in vitro apoptosis of SCNs was analyzed by a TUNEL assay (Figure 5A,B), immunocytochemistry against cleaved caspase 3 (Figure 5C,D), and flow cytometry using propidium iodide (PI) and Annexin V (Figure 5E,F), it was observed that the treatment of H_2_O_2_ induced the apoptosis of SCNs in vitro. Pretreatment of CM of ahNSCs (NSC #1) for 1 h before applying H_2_O_2_ significantly reduced the apoptosis of SCNs. It was observed that results of a Western blot (Figure 6A,B) and quantitative reverse transcription-polymerase chain reaction (RT-PCR) (Figure 6C,D) showing an increase in pro-apoptotic factor and Bcl-2-assocated X protein (BAX) and a decrease in anti-apoptotic factor and B-cell lymphoma 2 (Bcl-2), by H_2_O_2_ treatment, were reversed by the pretreatment of CM of ahNSCs (NSC #1). These results demonstrated that paracrine mediators contained in the CM of ahNSCs could inhibit neuronal apoptosis by mediating the neuroprotective effects of ahNSCs.

### 2.4. Neuroprotective Effects of ahNSCs Mediated by MCP-1

**Figure 7:**
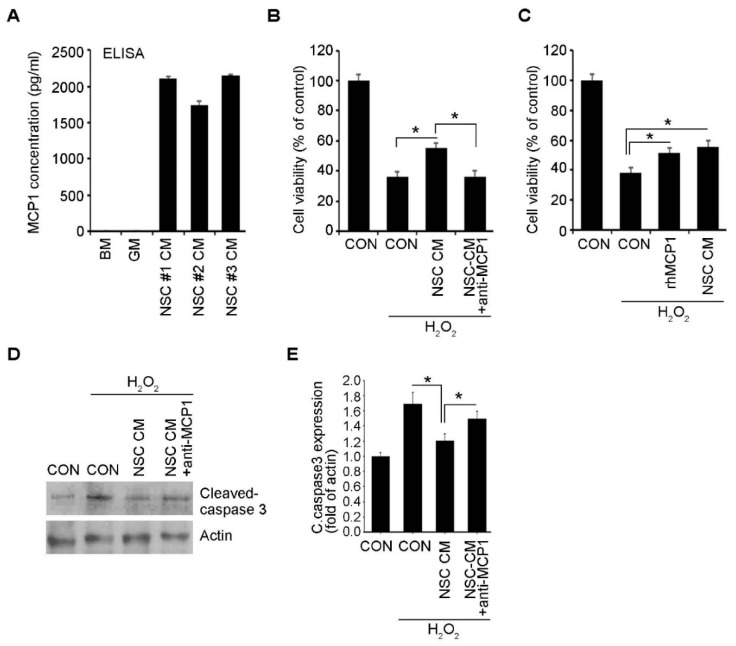
Neuroprotection effects of ahNSCs mediated by MCP-1. (**A**) The concentration of MCP-1 in the CM of three independent batches of ahNSCs was analyzed by ELISA. All experiments were performed in triplicate and repeated three times. (**B**) SCNs were treated with the CM of ahNSC #1 with/without the anti-MCP-1 neutralizing antibody (10 μg/mL) for 1 h and then with 200 μM of H_2_O_2_ for 6 h. Cell viability was analyzed by an MTT assay (*n* = 5 for each group). *, *p* < 0.05. (**C**) SCNs were treated with the CM of ahNSC #1 or recombinant MCP-1 protein (1 ng/mL) with 200 μM of H_2_O_2_ for 24 h. Cell viability was analyzed by an MTT assay (*n* = 8 for each group). *, *p* < 0.05. (**D**) SCNs were treated with the CM of ahNSC #1 with/without anti-MCP-1 neutralizing antibody (10 μg/mL) for 1 h and then with 200 μM of H_2_O_2_ for 24 h. The expression of cleaved caspase 3 was analyzed by a western blot analysis. Actin = loading ve. (**E**) The amount of cleaved caspase 3 was quantified by a densitometry analysis (normalized to actin) and compared (*n* = 3 for each group). *, *p* < 0.05.

To identify the paracrine mediator that shows the neuroprotective effects of ahNSCs, the analysis of various cytokines produced by ahNSCs was conducted [19]. The concentration of MCP-1 was found to be the highest among various types of paracrine factors in the CM of ahNSCs and was reported to have prominent neuroprotective effects [19]. A high concentration of MCP-1 in the CM of ahNSCs was confirmed by an ELISA assay in three different batches of ahNSCs (NSC #1, #2, and #3) (Figure 7A). Moreover, the in vivo production of MCP-1 in ahNSCs was confirmed in the rat brains by immunohistochemistry (data not shown). Based on the analysis, it was found that the presence of a MCP-1 neutralizing antibody to the CM of ahNSCs (NSC #1) offset and reversed the neuroprotective effects of ahNSCs (Figure 7B). In contrast, the presence of a recombinant protein of human MCP-1 (rhMCP1) showed neuroprotective effects similar to those of the CM of ahNSCs (NSC #1) (Figure 7C). In the western blot analysis, the treatment of the CM of ahNSCs also showed a significant decrease in the expression of cleaved caspase 3, which was reversed in the presence of an anti-MCP-1 neutralizing antibody in the CM of ahNSCs (Figure 7D,E).

## 3. Discussion

In the previous study, we demonstrated in vivo therapeutic effects of ahNSCs in SCI animal models, which were primarily isolated and cultured from surgical samples of the cerebral cortex with a hemorrhagic stroke [13]. These samples were obtained by the surgical process of removing some portion of the brain tissue of the cerebral cortex in order to lower the intracranial pressure of patients with stroke. In contrast, ahNSCs originated from the temporal cortex of FCD type IIIa were obtained and used in this study. FCD type IIIa is a type of FCD which has the combined abnormality of hippocampal sclerosis (HS) in the hippocampus and FCD in the cerebral cortex [20,21]. Although patients with FCD type IIIa suffer from epilepsy, the symptom has been reported to derive from pathologies of the hippocampus [22]. A histological analysis of the temporal cortex of FCD type IIIa could show abnormal cortical layering, such as microcolumns and dyslamination [23]. However, the abnormalities are localized and might be results of an epileptic seizure [24].

During surgery for hemorrhagic stroke and FCD type IIIa, cerebral cortex samples were obtained by decompression and an approach to the hippocampus, respectively, rather than for elimination of the disease focus [25,26]. Since these surgical samples harbor relatively normal histology, ahNSCs primarily cultured from the hemorrhagic stroke and FCD type IIIa shared very similar patterns of ahNSC-specific marker-expression, a differentiation potential to neural cells, and proliferation properties [14,15]. Moreover, when ahNSCs were transplanted into the brains of rodents, they did not induce any abnormal symptoms, including intracranial hemorrhage and seizure [13]. In SCI animal models, ahNSCs from both conditions demonstrated an identical relationship between the injection dose and treatment effects. There were differences in the treatment effects among the treated groups. The differences might have resulted from the number of ahNSCs that migrated into the lesions of spinal cords, which needs to be determined further. Our results indicated that ahNSCs from neurological disorders might be utilized to develop cell therapeutics for SCI.

Given the differentiation potential and paracrine factors of ahNSCs, ahNSCs might exert therapeutic effects for SCI via their differentiation into functional neural cells and excretion of neuroprotective paracrine factors [27,28,29]. Previously, the presence of human DNA in ahNSCs, which were transplanted into damaged spinal cords of rats, was observed for 5 weeks after transplantation by RT-PCR using human-specific primers. The amount of human DNA was found to be decreased gradually at the site of transplantation [13]. Regarding the fact that the functional recovery by ahNSCs transplantation in animal models of SCI has not been reduced as times goes on, the neuroprotective effects of ahNSCs for SCI could be mainly mediated by the paracrine factors of ahNSCs.

Our study demonstrates that the CM of ahNSCs as well as the transplantation of ahNSCs inhibits apoptosis of neural cells in oxidative stress and exerts neuroprotective effects in vitro and in vivo, respectively. Various types of cytokines were detected in the CM of ahNSCs [30,31]. Especially, the level of MCP-1 (CCL2) was relatively higher in the CM of ahNSCs. Since the concentration of MCP-1 was similar in three independent batches of CMs of ahNSCs, the high expression of MCP-1 might be one of key features of ahNSCs. Although MCP-1 is a well-known pro-inflammatory cytokine, its receptor, CCR2, is expressed by neurons in various locations of CNS, including spinal cord [32,33]. In neurons, MCP-1 reduced the ability of neuronal N-methyl-D-aspartate (NMDA) receptors to respond to ligands, thereby reducing glutamate release and subsequent neurotoxic effects [34]. In this study, the neuroprotective effects of the CM of ahNSCs on the survival of primarily cultured spinal cord neurons under the oxidative condition were offset and reversed by the presence of an MCP-1-neutralizing antibody. Since oxidative and excitatory stress are involved in the neuronal apoptosis in a damaged spinal cord [35,36], MCP-1 could be one of the major paracrine factors that mediate the in vitro and in vivo neuroprotective effects of ahNSCs. In vitro experiments in this study are not enough to explain the in vivo roles of MCP-1 in the treatment effects of ahNSCs. In vivo experiments using neutralizing antibodies against MCP-1 and/or specific shRNAs for MCP-1 should be required in future studies.

The presence of MCP-1 in a lesion of a damaged spinal cord attracts monocytes, which, in turn, could provoke local inflammatory reactions [37,38]. Conventionally, the inflammation in damaged neural tissue has been reported to worsen the destruction of tissue by diseases. However, recent studies have demonstrated that monocytes recruited in the lesion of SCI can differentiate anti-inflammatory/resolving phenotype macrophages and degrade the glial scar that prevents axons and dendrites from regrowth [39,40]. Although the results were not shown, a significant reduction of active astrocytes was observed in the high and medium group compared to the vehicle group in this study. A decrease in the glial scar formation by transplanting ahNSCs was reported in our previous study using ahNSCs from a hemorrhagic stroke in SCI animal models [13]. The effects of MCP-1 released by ahNSCs on the inflammatory reaction and glial scar formation in SCI animal models need to be elucidated further.

In conclusion, non-clinical results evidently show that ahNSCs from the temporal cortex of FCD type IIIa surgical samples were capable of exerting significant therapeutic effects for SCI, which were mediated by the neuroprotective paracrine factors of ahNSCs. Particularly, MCP-1 released by ahNSCs was considered to play an important role in mechanisms of neuroprotection and anti-apoptosis for SCI animal models. In this study, we set up stepping stones by providing promising non-clinical data which could encourage the future development of cell therapeutics for SCI, whereas there are no available therapeutic options at present.

## 4. Materials and Methods

### 4.1. Study Approval and Animal Care

Informed written consent was obtained from patients, according to the guidelines approved by the Institutional Review Board (IRB) of Samsung Medical Center (2016-11-085-003, Seoul, Korea). All animal studies were approved by the Institutional Animal Care and Use Committee (IACUC) from the Laboratory Animal Research Center (LARC) at Sungkyunkwan University (SKKUI-ACUC2018-05-06-3, Suwon, Gyeonggi-do, Korea). Animal experiments were conducted in accordance with the Guide for the Care and Use of Laboratory Animals from Institute for Laboratory Animal Research (ILAR) [41].

### 4.2. Primary Culture of Rat Spinal Cord Neurons (SCNs)

Spinal cords were isolated from E15.5 rat embryos and digested with 0.25% trypsin-EDTA (Gibco, Grand Island, NY, USA) at 37 °C for 2 min. The reaction was terminated using Dulbecco’s Modified Eagle’s Medium (DMEM, Corning, Corning, NY, USA) containing 1% penicillin/streptomycin (Gibco) and 10% fetal bovine serum (FBS, Gibco). Suspended cells were filtrated using 70-μm cell strainers (Corning) and then maintained in a neurobasal medium (Gibco) containing 2% B27 supplement (Gibco) and 1% penicillin/streptomycin at 37 °C in 5% CO_2_ humidified atmosphere. Half of the medium was replaced with fresh medium twice a week.

### 4.3. Primary Culture of ahNSCs

Three batches of ahNSCs (NSC #1, NSC #2, and NSC #3) were primarily cultured from the temporal lobes of donors with FCD type IIIa, as previously described [14]. Briefly, ahNSCs were maintained in DMEM: Nutrient Mixture F-12 Ham’s medium (DMEM/F-12, Gibco) containing 0.5% FBS, 2% B27 supplement, 1% penicillin/streptomycin, 20 ng/mL human epidermal growth factor (EGF, R&D, Minneapolis, MN, USA), and 20 ng/mL human basic fibroblast growth factor (bFGF, R&D) at 37 °C in a 5% CO_2_ humidified atmosphere. AhNSCs at in vitro passages 3–9 were used for the experiments. The differentiation of ahNSCs was induced in vitro, as previously described [14].

### 4.4. Collection of Conditioned Medium (CM) of ahNSCs

AhNSCs were seeded with a density of 9000 cells/cm^2^ on T75 culture flasks (SPL lifescience, Pocheon, Korea) and were cultured for 48 h. AhNSCs were washed 2 times with phosphate-buffered saline (PBS, Gibco) and maintained in DMEM/F-12 containing 1% penicillin/streptomycin for 24 h. The CM of ahNSCs was collected and centrifuged at 300 RCF for 3 min at 4 °C. The CM was stored at −80 °C.

### 4.5. Enzyme-Linked Immunosorbent Assay (ELISA) Assay

The concentrations of MCP-1 in the CM of three different batches of ahNSCs (NSC #1, NSC #2, and NSC #3) were analyzed by an ELISA kit for MCP-1 (Quantikine, R&D), according to the manufacturer’s instructions.

### 4.6. Cell Viability Assay

SCNs were incubated in 96- or 24-well culture plates (2 × 10^5^ cells/mL) at 37 °C in a 5% CO_2_ humidified atmosphere for 7 days. SCNs were treated with the CM of ahNSCs for 1 hr, washed with PBS, and then treated with a neurobasal medium containing 1% penicillin/streptomycin with/without H_2_O_2_ for 24 h. An anti-MCP-1 neutralizing antibody (10 μg/mL, Santa Cruz Biotechnology, Santa Cruz, CA, USA) and a recombinant MCP-1 protein (1 ng/mL, R&D) were applied and followed by adding 10 μL of 3-(4,5-dimethylthiazol-2-yl)-2,5-diphenyltetrazolium bromide (MTT, Sigma-Aldrich, St. Louis, MO, USA) into each well for 4 h. After 100 μL of dimethyl sulfoxide (DMSO, Panreac applichem, Barcelona, Spain) was added, the plates were incubated at 37 °C for 1 h. Light absorption was measured at a 595 nm wavelength. Cell viability was also evaluated by the trypan blue exclusion method using an automated cell counter (TC10, Bio-Rad, Hercules, CA, USA).

### 4.7. Western Blot

Cells were harvested and lysed using a RIPA lysis buffer (Santa Cruz Biotechnology) with a protease inhibitor cocktail (Roche, Indianapolis, IN, USA). An equal amount of proteins was separated by SDS-PAGE and analyzed by immunoblotting with primary antibodies for cleaved caspase3 (Cell Signaling Technology, Danvers, MA, USA), Bax, Bcl2, and actin (Santa Cruz Biotechnology).

### 4.8. Annexin V/Propidium Iodide (PI) Assay

Cells (1 × 10^6^ cells/mL) were stained with an Annexin V Apoptosis Detection kit (Invitrogen, Carlsbad, CA, USA), according to the manufacturer’s instruction, and then evaluated using flow cytometry.

### 4.9. Reserve Transcription-Polymerase Chain Reaction (RT-PCR)

RNA was isolated using a Takara MiniBEST Universal RNA Extraction Kit (Takara, Kyoto, Japan), according to the manufacturer’s instructions. The DNA-free RNA was reverse transcribed with a cDNA synthesis kit (Takara), according to the manufacturer’s protocol. Amplification was performed by denaturation at 95 °C for 5 min, followed by 35 three-step cycles of 95 °C for 30 s, 52 °C for 30 s, and 72 °C for 30 s. After amplification, PCR products were subjected to a 0.8–1.5% agarose gel electrophoresis and visualized by ethidium bromide. The primers used for amplification were as follows: BAX, forward 5′-GTGGCAGCTGACATGTTTG-3′ and reverse 5′-ATCAGCTCGGGCACTTTAG-3′; Bcl2, forward 5′-CTGAACCGGCATCTGCAC AC-3′ and reverse 5′-GCAGGTCTGCTGACCTCACT-3′; Actin, forward 5′-AGCCATGTACG TAGCCATCC-3′ and reverse 5′-CTCTCAGCTGTGGTGGTGAA-3′.

### 4.10. Immunofluorescence Analysis and Terminal Deoxynucleotidyl Transferase dUTP Nick End Labeling (TUNEL) Assay

Cells cultured on coverslips were fixed with 4% paraformaldehyde at 4 °C for 30 min. After washing with PBS, cells were permeabilized in PBS containing 1% Triton X-100 and 0.5% NP-40 (Sigma-Aldrich) and then blocked with 3% bovine serum albumin (BSA) and 1% normal horse serum (Santa Cruz Biotechnology). Cells were incubated with antibodies against Tuj1 (1:100, Abcam, Cambridge, UK), cleaved caspase3 (1:100, Cell Signaling Technology), and then appropriate secondary antibodies (Invitrogen). Cells were stained with a TUNEL kit (Roche, Indianapolis, IN, USA), according to the manufacturer’s protocol. 4′,6-diamidino-2-phenylindole (DAPI) (Vector Laboratories, Burlingame, CA, USA) was used for the nuclei. After mounting, the cells were examined using confocal laser scanning microscopy (BIORP, Leica, Wetzlar, Germany).

### 4.11. Spinal Cord Injury Animal Model

Ten-week-old Sprague-Dawley rats (female, Orient Bio., Sungnam, Korea) were anaesthetized with 2.5–5% isofluorane (Hana Pharm, Seoul, Korea). A 2 cm-long incision was made in the level of T9–T10 and, following laminectomy, exposed the thoracic spinal cord. Moderate SCI was induced by an Infinite Horizon (IH) impactor (200-kKdyn; Precision System Instrumentation, Fairfax, VA, USA). Cefazolin (33.3 mg/kg, Chong Kun Dang, Seoul, Republic of Korea) and ketoprofen (10 mg/kg, Uni Biotech, Seoul, Korea) were intramuscularly administered daily for 2 days to prevent infection and reduce pain. The urinary bladder was compressed twice daily until spontaneous micturition was observed. Motor function was measured by the Basso–Beatti–Bresnahan (BBB) locomotor rating test [42], weekly.

### 4.12. Cell Transplantation

Animals with a BBB score from 4 to 6 on the seventh day after SCI were randomly divided into four groups: the vehicle, low, medium, and high group (*n* = 8 for vehicle group and *n* = 10 for other groups). On the seventh day after SCI, the transplantation into the lateral ventricle (LV) was performed according to the previous report [43]. Briefly, rats were fixed in a rodent stereotactic device (Model 900 small animal stereotaxic instrument, KOPF stereotaxic, Tujunga, CA, USA) to make Burr holes (anterior–posterior (AP): −0.8 mm, medial–lateral (ML): 1.4 mm, dorsal–ventral (DV): 3.8). A total of 3, 10, or 30 × 10^5^ ahNSCs in 30 μL HBSS (Gibco) (low, medium, or high group, respectively) were injected into the LV for 10 min using a 26G Hamilton syringe (Hamilton Company, Reno, NV, USA) and a syringe pump (LEGATO™111, KD scientific, Holliston, MA, USA). A total of 30 μL HBSS was used for vehicle group. AhNSCs (NSC #1) at in vitro passage 6 (P6) were used. After transplantation, the injection needle was maintained for 5 min to prevent leakage and then removed at a speed of 1 mm/min. A total of 10 mg/kg ketoprofen was administrated intramuscularly to reduce pain after the surgery. For immunosuppression, 10 mg/kg cyclosporine A (Chong Kun Dang Pharmaceutical Corp., Seoul, Korea) was subcutaneously administered daily from 24 h before transplantation to sacrifice.

### 4.13. Immunohistochemistry and TUNEL Assay

Spinal cords of animals with SCI were embedded in paraffin, as described previously [16]. Formalin-fixed, paraffin-embedded tissues were sectioned (4 μm) and then placed on silane-coated slides (Muto Pure Chemicals Co., Ltd., Tokyo, Japan). After heating on a slide warmer (Lab-line Instruments USA, Dubuque, IA, USA) at 65 °C for 30 min, slides were deparaffinized and rehydrated. Antigen retrieval was carried out by boiling in the target retrieval solution (Dako, Carpentaria, CA, USA) (4 times, 5 min for each). Slides were incubated in 0.3% ammonia in 70% methanol for 1 h, washed in 50% methanol for 10 min, and then incubated in 3% hydrogen peroxide in methanol for 12 min to quench the endogenous peroxidase activity. Slides were incubated in a peroxidase-blocking solution, including 5% BSA and 2% normal goat serum (GenDEPOT, Katy, TX, USA) in a peroxidase-blocking solution (Dako), for 1 h at RT. Primary antibodies were then added and incubated at 4 °C overnight: NeuN (1:500, Millipore, Temecula, CA, USA), Tuj1 (1:1000, Abcam), and cleaved caspase 3 (1:50, Cell Signaling Technology). Sections of the spinal cord were incubated with an appropriate Horseradish peroxidase (HRP)-conjugated secondary antibody (Abcam) for 1 h at RT, visualized by 3,3′-diaminobenzidine tetrahydrochloride (DAB, Dako), and then counterstained with hematoxylin (Sigma-Aldrich). For immunofluorescence, sections were treated with appropriate secondary antibodies and then counterstained with DAPI. Paraffin sections were stained with a TUNEL kit (Roche), according to the manufacturer’s protocol.

### 4.14. Image Analysis

For DAB staining, colors in the scanned images were separated into purple and brown using the QuPath image analysis software (Queen’s University Belfast, Northern Ireland, UK), as described previously [13,44]. A computational color deconvolution was applied to separate DAB staining from the hematoxylin. The brown immunohistochemical staining was analyzed using ImageJ software (NIH Image, Bethesda, MD, USA). For immunofluorescence, nuclei with positive signals were counted in each section. In the western blot results, bands were quantified using image J software and normalized to actin.

### 4.15. Statistical Analysis

In vivo and in vitro data were shown as the average ± standard error (SEM) and the average ± standard deviation (SD), respectively. A *p*-value < 0.05 in the two-tailed Student’s *t* test was interpreted as statistically significant.

## Data Availability

The data presented in this study are available on request from the corresponding author.

## Supplementary Materials

The following supporting information can be downloaded at: https://www.mdpi.com/article/10.3390/ijms23084267/s1.
