# Supplementary material for: Significant Therapeutic Effects of Adult Human Neural Stem Cells for Spinal Cord Injury Are Mediated by Monocyte Chemoattractant Protein-1 (MCP-1)
Source: Int J Mol Sci. 2022 Apr 12;23(8):4267. doi: 10.3390/ijms23084267 (PMC9029183; doi:10.3390/ijms23084267)
Supplement: Supplementary file 1 [file ijms-23-04267-s001.zip › ijms-1621991-supplementary.pdf]

Supplementary Material

Significant Therapeutic Effects of Adult Human Neural Stem Cells for Spinal Cord Injury Are Mediated by Monocyte Chemoattractant Protein-1 (MCP-1)

Table S1. BBB score [1].

Group	No. of Animal	1WK	2WK	3WK	4WK	5WK	6WK
Vehicle	8	5.4 ± 0.52	8.8 ± 0.71	8.9 ± 0.83	9.3 ± 0.87	9.5 ± 1.41	9.3 ± 0.89
Low	10	5.2 ± 0.67	9.3 ± 1.16	9.4 ± 0.84	10 ± 0.94	10.4 ± 1.07	10.7 ± 0.82 *
Middle	10	5.3 ± 0.54	10.3 ± 0.82 *	10.7 ± 1.06 *	11.5 ± 0.85 *	11.8 ± 0.79 *	11.8 ± 0.63 *
High	10	5.3 ± 0.63	9.8 ± 0.79 *	10.4 ± 0.97 *	10.9 ± 1.20 *	11 ± 1.25 *	11.2 ± 1.48 *

[1] Average ± Standard Error (SEM), * $p < 0.05$ vs. Vehicle.

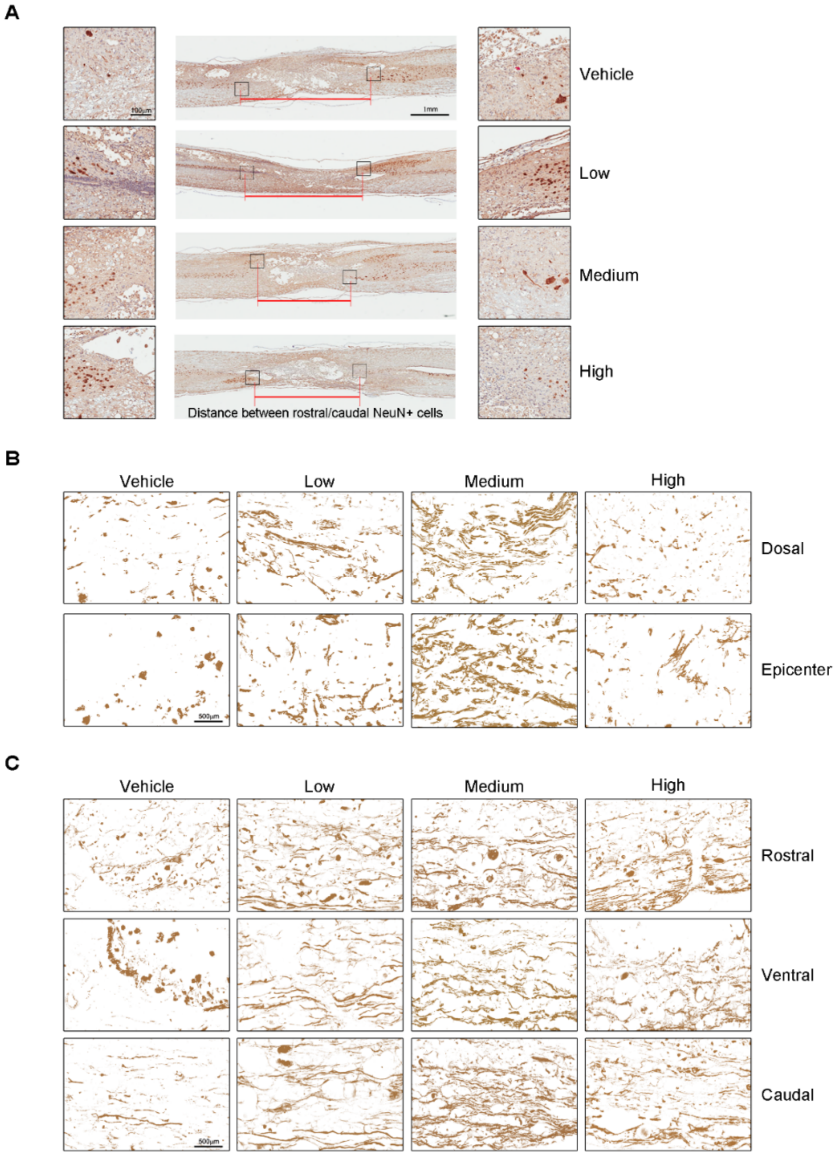

Figure S1. Immunohistochemistry against NeuN and Tuj1. (**A**) NeuN-positive neurons adjacent to damaged lesions both rostrally and caudally were identified. The distance between those neurons were measured (read lines). (**B**) Tuj1 immunoreactivity was evaluated in the areas of damaged spinal cord: Dorsal and Epicenter. (**C**) Tuj1 immunoreactivity was evaluated in the areas of damaged spinal cord: Rostral, Ventral, and Caudal.
